# Microfinance towards micro-enterprises development in rural Malaysia through digital finance

**DOI:** 10.1007/s43621-021-00066-3

**Published:** 2021-12-06

**Authors:** Muhammad Farhan Jalil

**Affiliations:** School of Business and Management, University College of Technology Sarawak, No. 1, Jalan Universiti, 96000 Sibu, Sarawak Malaysia

**Keywords:** Microfinance, Micro-credit, Micro-savings, Micro-insurance, Digital finance, Micro enterprises (ME), Bottom of the pyramid

## Abstract

Microfinance is critical for the development of micro-enterprises and alleviating poverty. However, micro-enterprises are able to get microfinance services, they would face a variety of obstacles, due to the misunderstandings among many stakeholders, microfinance has not acquired widespread acceptance. Therefore, the purpose of this study is to investigate microfinance's impact on the sustainable development of Malaysia’s rural micro-enterprises. Besides, digital finance is integrated into the conceptual model to further investigate their mediating impact. Data was collected from 563 rural micro-enterprises using structured questionnaires, which were then statistically analyzed using AMOS-21. The findings of the study reveal that microfinance has a positive substantial influence on rural micro-enterprises development. Moreover, digital finance partially mediates the relationship. Thus, the study concludes that microfinance institutions are needed to adopt digital finance to enhance micro enterprises’ productivity through low transaction costs. The findings of the study can be useful to policymakers in the micro-enterprise sector who have a long-term vision and expect the sector to develop steadily. The study also provides scope and space for future academics and scholars to conduct further research.

## Introduction

Malaysia is one of the fastest growing economies in the world, but the majority of its small business owners remain impoverished [42]. One of the most widely held beliefs in the bottom of the pyramid (BOP) studies for decades has been that impoverished people would always have restricted access to financial services [10]. The BOP term referring to a segmenting the market in which clients have a yearly income of less than $1500 [5]. Prahalad [68] referred “people from BOP as successful customers”. Kolk et al. [48] also argued that identifying the poor is the most effective approach to reduce poverty. A market-based approach to poverty reduction is provided by the BOP viewpoint [47]. The BOP theory provides a comprehensive and effective framework for understanding the characteristics of micro-enterprises (MEs) in Malaysia. Successful BOP initiatives help MEs to develop more sustainably through microfinance services [71]. The adoption of digital finance within the BOP sector is the most serious impediment to the poor into long-term development [5]. Nevertheless, there is indeed a long-held assumption that MEs are resistant to financial technology adoption [27]. Baishya and Samalia [17], on the other hand, argued against this conventional wisdom, claiming that there is a high need for financial technology in the BOP market. As, MEs have access to a mobile banking through phones, allowing them to participate more actively in digital finance [67]. Furthermore, according to Murphy [58], the complexity of an innovation has a substantial influence on its acceptance in the BOP sector. As a result, it's critical to comprehend ME behavior in Malaysia's BOP microfinance sector. Economic studies have discovered that the economic development of MEs and the financial sector are both impacted by social variables [1]. The incorporation of BOP theory increases the study's primary emphasis. Therefore, this research presents the microfinance services towards sustainable developemnt of MEs in Malaysia. Digital finance was also included as a modifying component to explore its effects on MEs’ perceptions on services of microfinance.

It is indeed worth noting that BOP marketplaces cover a wide range of segments, including impoverished rural populations, poor entrepreneurs, and landless people [83]. In this respect, rural micro enterprise (ME) owners can be classified as a sector of Malaysia's BOP market. Such an assumption is reasonable, given that the majority of micro business owners are impoverished, have poor infrastructure, and have less resources [56].

A growing number of businesses are focusing on the potential commercial prospects in the BOP market by developing services that cater solely to the poor [36]. Microfinance, for example, meets the financial needs of clients in the BOP markets in a straightforward manner [71]. Microfinance is a sort of financial paradigm in which financial firms offer a range of high-quality financial services to everyone, including poor entrepreneurs [21]. When compared to typical financial companies, microfinance institutions' main goal is to make economical financial services more accessible to underprivileged clients [40]. As a result, microfinance is critical for alleviating poverty through fostering the development of rural MEs.

The access to finance is most important for the rural entrepreneurs’ growth and development [38]. MEs required different sources of financial help to get eligible outcome but it always been unable to access such funds from anywhere because of their improper financial statement, limited assets or savings and lack of developed business plans [40]. Banks or commercial institutions only entertained those who are already established in their area and access to credit are very easy for them. The lack of credit access makes it more crucial for rural MEs to manage their allocated funds, invest efficiently and produce effective outcomes [80]. Most micro enterprises used internally generated fund as source of financing their businesses and external source is hard to find which may cause their failures [33]. However in Malaysia, according to Muridan and Ibrahim [57], 33% of small and 51.7% of medium enterprises had used external financing from microfinance institutions compared to only 17.4% for micro enterprises. Moreover, 50% rural MEs stopped their operation in the beginning for 3 to 5 years and the rate of start-up level of such enterprises also decreased [57].

Microfinance is a large sector in Malaysia with a substantial impact on the economy [8]. However, the MEs faces several problems, including credit risk and a lack of knowledge of emerging technological potential [31]. These issues may be overcome by industry practitioners utilizing developing digital innovations like digital finance to enhance their financial efficiency [67]. The potential to improve microfinance services by utilizing new digital financial technology are highlighted by Mushtaq and Bruneau [59]. Hence, Malaysian microfinance institutions must incorporate developing digital finance to provide cost-effective services to rural MEs. Furthermore, digital finance is a new technology that has sparked a lot of interest in recent years, but there is still a wide range of research to be conducted and a massive effort to be done. This research fills the gap by determining the current state of digital finance adoption in Malaysia's microfinance industry in order to assist the development of rural MEs.

However, the development of MEs in Malaysia’s rural areas is fraught with both problems and possibilities [2]. Is it possible for microfinance, particularly financial services, to play an important role in the development of rural MEs? Is it possible to use digital finance to mediate the relationship between microfinance and the development of rural MEs? These highlighted concerns require more examination by researchers, as well as additional effective methods to improve microfinance services for the development of rural MEs in Malaysia.

Microfinance services play a critical role in the growth of MEs, and their absence may threaten the sector's long-term viability. However, the impact of microfinance services on rural ME development is questionable. Ul-Hameed et al. [82] try to explore the impact of microfinance on ability of low-income households to start new businesses by undertaking a systematic review, while Banerjee et al. [18] discovered a beneficial association between microfinance programmes and business growth. Babajide [15] find the effect of microfinance on the poor, focussing on the sub-Saharan region. In the ME sector, development has received minimal attention, particularly in Malaysia's rural areas. More importantly, the impact of microfinance on the long-term development of MEs has received little scholarly attention. Previous research [12, 47] has emphasized the relevance of digital finance, but its influence on ME development has yet to be investigated. Given the positive relationship between digital finance and microfinance, MEs must be able to deal with and adapt to change in order to survive and thrive. However, further research into the relationship between microfinance services, digital finance, and ME development is required. Therefore, the focus of this study was on microfinance services to support ME development. It also focuses on the role of digital finance to strengthen the relationship between microfinance services and ME’s development in rural areas. Furthermore, this research is essential for microfinance institutions, Malaysian policymakers, and other practitioners who want to assist ME sector. With various services given by microfinance institutions, this study is also useful to the elimination of poverty among Malaysian poor entrepreneurs.

Following the introduction, the remainder of the article is arranged as follows: Sect. 2 reviews relevant literature and empirical studies, and proposes research hypotheses and a conceptual model; Sect. 3 describes sample data, and methods; Sect. 4 explains the results of a study; Sect. 5 discusses the findings and presents theoretical and policy implications; and Sect. 6 describe conclusion of the study, as well as its limitations and future directions.

## Literature review

### Microfinance

Microfinance has evolved into a thriving business throughout the world’s BOP market in recent years, and it is the quickest growing sector, where venture capital funding and risk are important [61, 71]. Microfinance began in Bangladesh in 1976, when its founder, Nobel Laureate Professor Muhammad Younus, established the first microfinance organization in the country following successful experiments of lending small amounts of money to underprivileged residents in Bangladesh villages [52]. This journey aided their micro enterprises development, standard of living, developing skills, wealth, and improvement in economic empowerment tremendously [70].

Malaysia's microfinance services has been in place since 1987 as one of the country's poverty-reduction strategies. Amanah Ikhtiar Malaysia (AIM) and The Economic Fund for National Entrepreneurs Group (TEKUN) are two significant microfinance institutions in Malaysia [14]. AIM and TEKUN are poverty-focused organizations that exclusively give microcredit loans to Malaysians that are poor or have a low income. Since its inception, each of these microfinance institutions has had its own lending structure and has been subsidized by the government [54]. Furthermore, microfinance institutions all governed under the government regulation [85]. Therefore, these institutions are under the control of Bank Negara Malaysia (BNM), and all are operate on a same platform provided by the BNM.

Services provided by microfinance institutions plays an important role in the development of Mes. The consequences of microfinance services on ME developmental processes should be articulated. For instance, microfinance services helped in the expansion of businesses and the creation of more jobs and placed optimistic impression on the development of micro and small business [25, 43]. The previous studies [15, 19] found microfinance services assisting clients in starting new businesses had a favourable impact on ME growth and increased business activities. Clients of microfinance programmes experienced a growth in their microbusiness [40]. Similarly, Ul-Hameed et al. [82] discovered that microfinance services have a favourable impact on the growth of businesses and the ability of low-income households to start new businesses. Furthermore, Banerjee et al. [18] discovered a beneficial association between microfinance programmes and business growth. Thus, the hypothesis are;

**H1**: Microfinance has a significant impact on the development of MEs.

### Micro-credit

Microfinance has the ability to improve the economic well-being of poor entrepreneurs in the BOP market. Microfinance services are an important tool for reducing, and eliminating poverty, and generating wealth for poor entrepreneurs [41]. The three main financial services offered by the microfinance programme are micro-credit, micro-savings, and micro-insurance. These services are often recognized as necessary for business growth, and development [40].

Micro-credit is the most well-known service provided by microfinance programmes in the BOP market. The most important aspect of MEs is credit [3, 4]. According to the current literature [51, 63, 78], a suitable quantity of credit improves the performance of entrepreneurial activities. Moreover, credit helps poor entrepreneurs, because it is well acknowledged that credit improves productivity, investment, and income [16, 49]. Therefore, it raises the success rate of MEs, which helps to ensure their sustainability in BOP market. Micro-credit and micro-enterprises have also been proven to have a strong beneficial relationship in several research [26, 40]. It also improves business dedication, which boosts satisfaction and motivation [51]. As a result, micro-credit is one of the most important factors in the sustainable development of Mes [45, 64]. As a result of the discussion, it can be stated that micro-credit is one of the most important microfinance services for MSE development. Thus, it is hypothesized that:

**H1a**: Micro-credit is an influential financial service of microfinance for the development of MEs.

### Micro-savings

Another financial service provided by microfinance programmes are micro-savings, which allows BOP market participants, particularly poor entrepreneurs throughout the world, to save some money on monthly basis. Entrepreneurs can use their savings to obtain a loan that is based on their savings, allowing them to increase productivity of their enterprises [73]. Therefore, savings are beneficial in increasing entrepreneurial activities of poor entrepreneurs [40]. According to Ul-Hameed et al. [82], both savings and credit have a substantial impact on enterprise growth and performance in Pakistan. Furthermore, according to a research done in Tunisia, savings as a service provided by microfinance institutions has a favorable impact on MSE sustainability [55]. Therefore, saving is an important service of microfinance for the development of MEs. Thus, the hypothesis as follows:

**H1b**: Micro-savings is an influential financial service of microfinance for the development of MEs.

### Micro-insurance

Microinsurance is another financial service provided by microfinance firms. Micro-insurance protects BOP market entrepreneurs against particular risks in return for monthly premium payments equal to their source of survival, which has a risk cost [72]. Micro-insurance involves the acquisition of assets for enterprises with insurance coverage, yet many commercial banks overlook this service [7]. Since insurance is one of the defenses against every unanticipated risks, not having insurance raises the risk of using microcredit [18]. MSEs have a better chance of succeeding if they have insurance coverage. In Pakistan, according to Hameed et al. [40], insurance is one of the tools that helps to alleviate the consequences of risk. MSEs have a better chance of succeeding if they are better protected against major disasters. Microfinance services, according to Olaosebikan and Adams [65], should provide different services, including insurance, to assist in the development of successful entrepreneurs. Therefore, micro-insurance is one of the solutions available to protect MEs from any unforeseen circumstances and it improves the long-term sustainability of ME. Thus, the hypothesis as follows:

**H1c**: Micro-insurance is an influential financial service of microfinance for the development of MEs.

### Digital finance

According to the Aisaiti et al. [5], digital finance refers to new business models in the financial industry that are fueled by large artificial intelligence, datasets, cloud computing, and other cutting-edge technology. Microfinance's major objective is to expand consumer breadth, availability, and satisfaction of services in order to satisfy the growing financial needs of poors in the BOP market [23], including MEs, to acquire appropriate services at a reasonable interest rate in a simple, effective, and safe way [30, 74]. Artificial intelligence, the internet, cloud computing, and big data have all become more popular academic topics, resulting in the emergence of a new idea known as Fintech [12]. Fintech is not only a mix of finance and technology; it also refers to the use of different technological techniques to increase service quality and, as a result, save operational expenses [47]. Transaction costs are reduced with digital finance because time and human resources are saved. As a result, it is critical to consider the importance of innovation and digital finance in promoting the sustainable development of MEs and microfinance in the digital era.

Microfinance's major objective is to expand the scope of MEs [40] and provide financial services that fulfil the growing financial demands of BOP market consumers in a simple, efficient, and safe manner [5]. Digital finance plays a key role in enhancing service efficiency and saving a large amount of processing costs incurred by using traditional financial services [47]. Furthermore, by offering easy and flexible financial services, digital finance has significantly reduced transaction costs and increased microfinance accessibility [60]. Connectivity between suppliers and customers, as well as between customers and customers, is a key aspect of digital financial services. Customers can use their mobile phones to access and interact with the supplier [67]. Micro enterprises may easily access funds for enterprise innovation and creativity due to digital financing [37]. Moreover, in terms of sustainable economic development, vulnerable MEs rely more heavily on digital financing, and they generate higher marginal revenue by using microfinance [62]. Moreover, with lower transaction costs and faster turnaround times, digital finance has the potential to strengthen the relationship between financial services and ME sustainability [59]. Unlike traditional face-to-face transactions, digital finance allows MEs to move funds via their digital account even without risk of losing currency notes [22]. Additionally, digital finance's efficiency and electronic transparency may reduce MSEs' risk perceptions. Therefore, the hypothesis as follows:

**H2**: Microfinance has a significant relationship with digital finance.

**H3**: Digital finance has a significant relationship with ME devevlopment.

**H4**: The mediating effect of digital finance between microfinance and ME development.

### Research framework

**Fig. 1:**
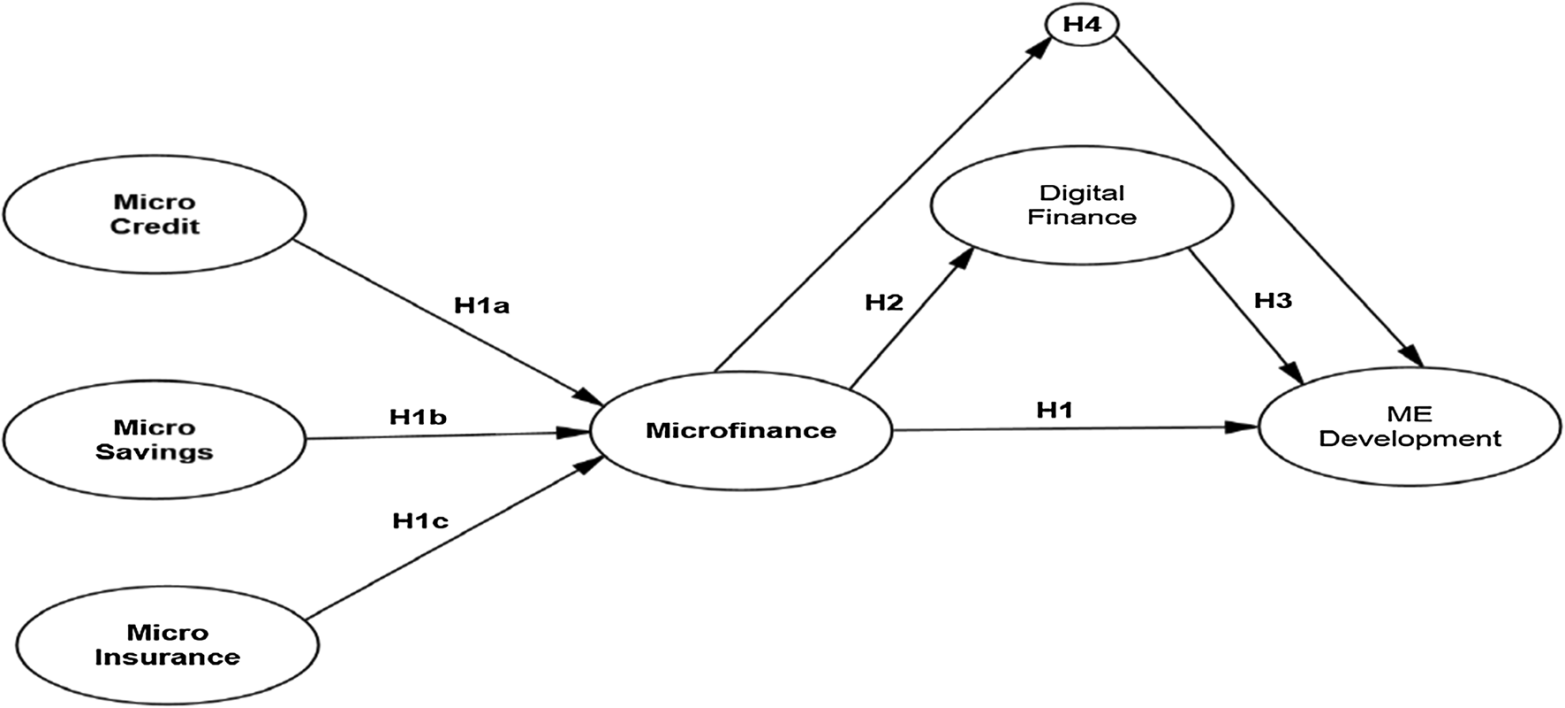
Conceptual framework

As previously discussed, it is critical to comprehend microfinance services and rural MEs development in Malaysian BOP market. The incorporation of BOP theory increases the research's main emphasis. Thus, Fig. 1 shows the conceptual model for this study, which represents microfinance services and their impact on the sustainable development of MEs in Malaysia. The major microfinance services were conceptualized using this paradigm. Digital finance was also introduced as a mediating component to explore its effects on microfinance and ME development.

### Methodology

Venkatesh et al. [84] argued that the methods used to lead the research must be on the same path as the main objective. Hence, a quantitative method was carried out in this study to test the hypotheses and to achieve the aim of the study. According to Queirós et al. [69] quantitative approaches are structured method for uniting inferential reason with defined empirical investigations in order to find and verify a set of probabilistic causal relationships. Similarly, according to Smith and Hasan [76], relating a quantitative approach to support the scholars to created statistical proof on the depth of relationships among both dependent and independent constructs.

### Model

#### Statistical analyses

The data acquired via questionnaire was evaluated using structural equation modelling (SEM). SEM is a statistical method used AMOS 21.0 to perform confirmatory factor analysis and path analysis. In the first step, confirmatory factor analysis (CFA) was used to test the measurement model's convergent validity and causal connection among modified items and variables [24, 39, 46]. In the second stage, the structural model was used to assess the relationship between the exogenous variables (microfinance services) and the endogenous variables (digital finance and ME development) [35]. In addition, the sobel test and bootstrap approach were used, which is considered as a good tool for evaluating the mediating influence of the construct in challenging circumstances [28]. SEM is the preferred method for testing causal relationships between constructs with multiple measurement items as well as empirically examining the model of the study.

### Instruments of the study

The selection of items to measure the microfinance services (micro-credit, micro-savings, and micro-insurance), MEs development, and the mediating effect of digital finance were chosen from the literature that is based on MEs’ perception. This study adapted items from previous studies with reliable and valid items used to measure the constructs. Items for measuring microfinance (micro-credit, micro-savings, and micro-insurance) were adapted from Hameed et al. [40], and Babajide [15]. To measure the digital finance, items were adapted from Aisaiti et al. [5]. Similarly for the construct of ME development, the items adapted from Kisaka and Mwewa [44], Babajide [15], and Bekele and Worku [20].

### Data sample

#### Data collection procedure and sample

Data can be collected in a number of ways and sources such as self-administered questionnaires, telephone interviews, and personal interviews. The form of the self-administered questionnaire used for this study is also known as the “drop-off survey”. This technique requires the researcher or representative of the researcher (i.e., enumerators in this study) to move to the respondent’s location and deliver questionnaires to respondents [77].

A study based on Structural Equation Modeling (SEM), the sample size would be at least in the range of 100 to 200 [46]. Others have recommended the lowest sample size of 200 cases [79]. Data collected from the different rural districts of Sarawak and Sabah in Malaysia from November 2020 to March 2021, adopted the stratified random sample method. The number of respondents set according to the total number of MEs registered in SMEs Crop Malaysia. Contacted 785 respondents and after screening the data, only 563 (71.7%) questionnaires appeared to be completed in all respects for further analysis. Meanwhile the percentages of the missing items were less than 5% and therefore, were replaced through the mean replacement procedure [29].

### Specification of micro, small, and medium enterprises

Micro, small, and medium enterprises (MSMEs) are conceptualized in a variety of ways by various authors and differs from country to country [9, 32]. Hence, MSMEs in one region may not be classified as in another. Some countries define MSMEs in terms of total assets, capital investment, and labor force employed, while others define MSMEs in terms of total assets, capital investment, and workforce. In the Philippines, MSMEs are defined by two operational definitions: employment and total assets [13]. Moreover, MSME definitions in Nigeria are based on revenue and employees [6]. Total assets, number of employees, yearly turnover, and capital investments are all factors that are used to define MSMEs in different nations. For the purposes of this study, Table 1 presents the SME Crop Malaysia definitions of MSMEs.

**Table 1:** Classification adopted by SME Crop on MSMEs in Malaysia

Size category	Employment	Assets (RM million)
Micro enterprises	Less than 5 employees	Less than RM 0.25
Small enterprises	Between 5 and 50 employees	Between RM 0.25 and less than RM 10
Medium enterprises	Between 51 and 150 employees	Between RM 10 and less than RM 25

## Results

### Sample characteristics

The demographic characteristics of the respondents through survey questionnaires from different states in Malaysia. The respondents’ profile has been classified based on their gender, age, marital status, education, religion, income level, and number of employees. Based on the notion of MEs is dependent on the number of employees. Thus, MEs with more than five employees were removed from the study. According to the items in the questionnaire, a descriptive statistic was used to perform a demographic analysis to determine the respondents' backgrounds as shown in Table 2.

**Table 2:** Personal profile of respondents

Variables		Number	Percentage
Gender	Male	344	61.1%
	Female	219	38.9%
Age	Less than 25	53	9.4%
	26–35	89	15.8%
	36–45	186	33.0%
	45–55	157	27.9%
	Above 55	78	13.9%
Marital status	Single	94	16.7%
	Married	339	60.2%
	Widow	43	7.6%
	Divorced	87	15.5%
Education	High school or less	387	68.7%
	Diploma	119	21.1%
	Bachelor degree	49	8.7%
	Master or above	8	1.5%
Religion	Muslim	268	47.6
	Hindu	66	11.7
	Christian	98	17.4
	Buddhist	110	19.5
	Others	21	3.8
Monthly income level	Less than RM1500	496	88.0%
	RM1500 or above	67	12.0%

### Normality statistics

As one of the assumptions of SEM that all the samples were tested for normality to assure data quality and accurate study outcomes [66]. Mishra [53] identified that skeweness and kurtosis have been used for determining the normality of the data. Therefore, in this study the skewness and kurtosis have been used for each construct, generated results show that the skewness and kurtosis were inside the acceptable range of the ± 3 (see Table 3).

**Table 3:** Descriptive statistics

Constructs	Range	Mean	Std. dev	Skewness	Kurtosis
Micro-credit	1–7	4.35	0.32	− 0.371	0.165
Micro-saving	1–7	4.79	0.39	− 0.091	0.328
Micro-insurance	1–7	4.87	0.36	− 0.543	0.604
Digital finance	1–7	4.33	0.41	− 0.496	− 0.297
MSE development	1–7	4.64	0.44	− 0.108	0.171

### Confirmatory factor analysis (CFA)

Confirmatory factor analysis (CFA), the first step of two-stage SEM statistical method, allows the researcher to rectify measurement error during the assessment of multiple variable relationships [66]. Maximum likelihood valuation is used to estimate the associations between the variables and their corresponding indicator items. Previous studies [53, 75] identified that, in CFA each item's factor loading should be 0.60 or above to be considered acceptable. According to the findings, the factor loading of items and fit indices were in the acceptable range, summarized CFA results as shown in Table 4.

**Table 4:** Results after CFA

Constructs	Chi-Square	df	CMIN/df	GF1	AGFI	CFI	RMESA
Micro-credit	20.825	4	2.099	0.976	0.938	0.984	0.072
Micro-saving	19.674	4	2.983	0.974	0.938	0.982	0.075
Micro-insurance	21.791	4	2.955	0.969	0.907	0.967	0.069
Digital finance	22.027	3	2.445	0.977	0.932	0.989	0.078
ME development	21.080	5	2.210	0.970	0.921	0.982	0.076

### Overall measurement model

**Fig. 2:**
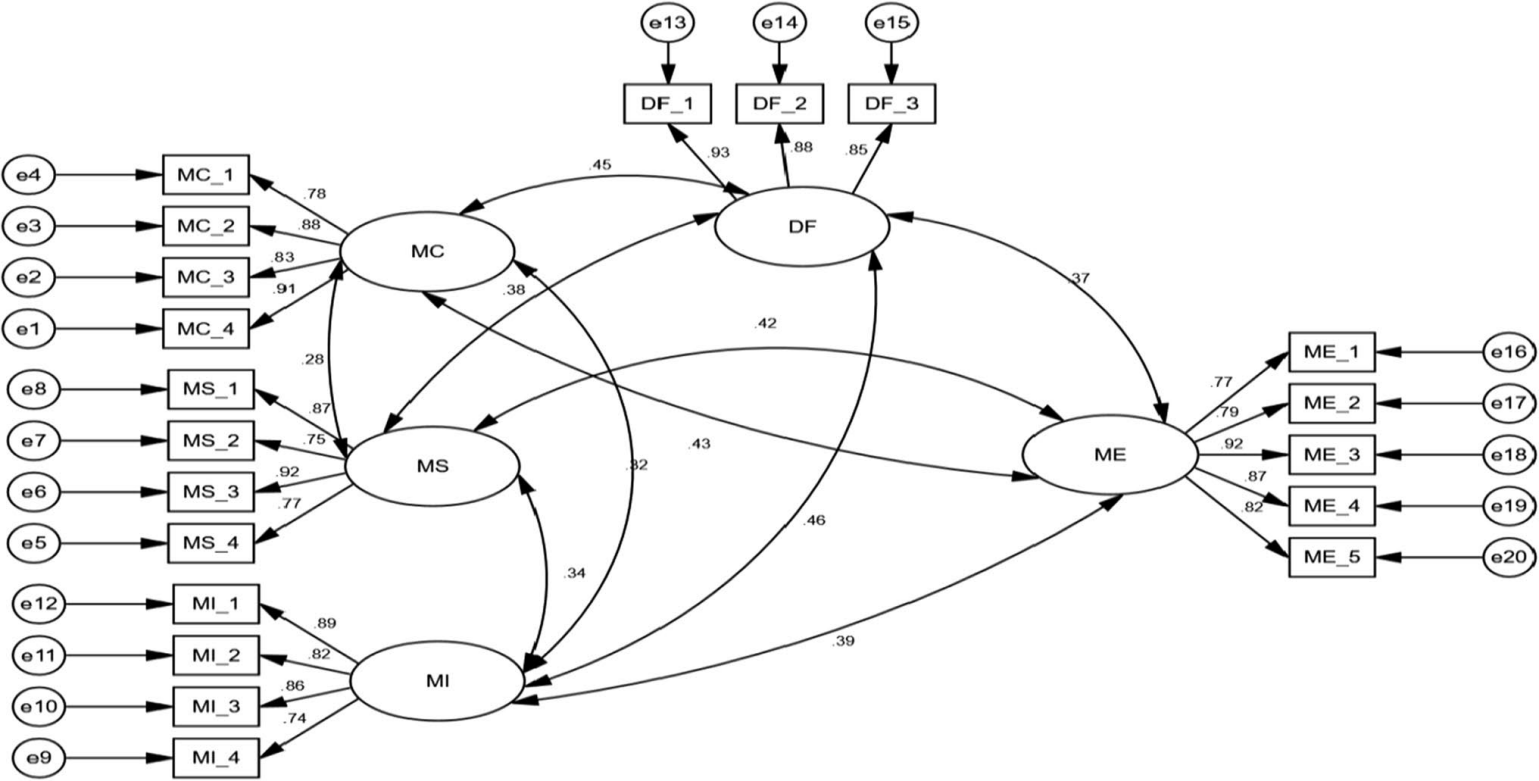
Measurement model

The overall measurement model was examined after the CFA validation results. The model that links the latent constructs to its indicators is referred to as the overall measurement model [66]. The results of this investigation showed that the overall measurement model's goodness-of-fit indices were well-fitting, such as RMSEA of 0.033 and chi square value of 527.54 with 562 degrees of freedom, GFI = 0.931, AGFI = 0.936, CFI = 0.952, and CMIN/df = 1.787. Fit indices were in the acceptable range as identified by (Castro-Jiménez et al., 2020). Figure 2 depicts the overall measurement model.

### Average variance extracted (AVE) and composite reliability (CR)

The findings of the study show that all of the constructs' Cronbach's Alpha (α) and Composite Reliability (CR) values are over 0.7, indicating that variables are internally consistent. Furthermore, the value of average variance extracted (AVE) for all variabless was found to be greater than 0.5, indicating that the constructs are convergent valid [34].

As described by Fornell and Larcker [34], “the discriminant validity was determined by comparing the square root of each AVE in the diagonal with the correlation coefficients (off-diagonal) for each construct in the relevant rows and columns”. The square root of the average variance extracted (AVE) value in this study is more than 0.6 for all constructs. Table 5 shows the average variance extracted (AVE) and composite reliability (CR) findings.

**Table 5:** Measurement model evaluation

	Items	Standardized Loading	α	CR	AVE
Micro-credit	MC_1	0.78	0.876	0.913	0.725
	MC_2	0.88			
	MC_3	0.83			
	MC_4	0.91			
Micro-saving	MS_1	0.87	0.833	0.898	0.690
	MS_2	0.75			
	MS_3	0.92			
	MS_4	0.77			
Micro-insurance	MI_1	0.89	0.839	0.898	0.688
	MI_2	0.82			
	MI_3	0.86			
	MI_4	0.74			
Digital finance	DF_1	0.93	0.882	0.917	0.787
	DF_2	0.88			
	DF_3	0.85			
ME development	ME_1	0.77	0.891	0.920	0.699
	ME_2	0.79			
	ME_3	0.92			
	ME_4	0.87			
	ME_5	0.82			

### Structural model

**Fig. 3:**
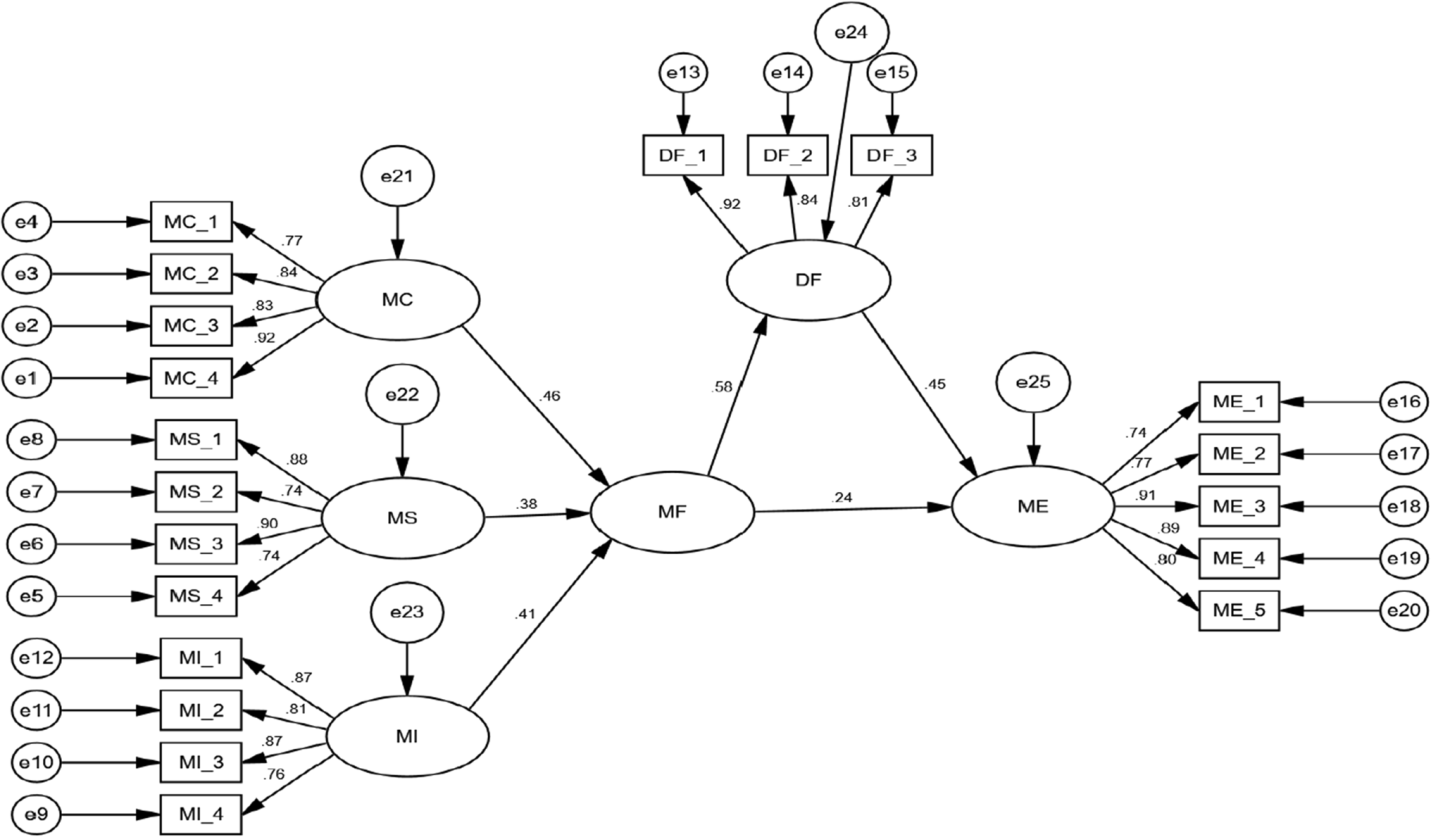
Structural model

The structural model (stage 2) was used to investigate the relationship of microfinance, digital finance and micro-enterprises development. AMOS 21.0 was used to evaluate the data. In contrast to earlier studies [39, 46, 66], the goodness-of-fit indices are tested in this study. As shown in Fig. 3, structural model of the study were well-fitted with RMSEA of 0.034 and a chi square value of 574.352 with 562 degrees of freedom, GFI = 0.928, AGFI = 0.917, CFI = 0.946, and CMIN/df = 1.896.

The findings of direct relationships are shown in Table 6. A level of significance for p-value < 0.05 as recommended by Kline [46], was also taken into account in the study. In assessing the hypothesized relationship, the hypotheses H1, H1a, H1b, H1c, H2, and H3 were statistically significant.

**Table 6:** Testing direct relationship

Paths	ß	Z-value	P-value	Significant
H1: MF– > MED	0.24	3.977	0.007	Yes
H1a: MC– > MF	0.46	5.492	***	Yes
H1b: MS– > MF	0.38	4.725	***	Yes
H1c: MI– > MF	0.41	5.018	***	Yes
H2: MF– > DF	0.58	6.189	***	Yes
H3: DF– > MED	0.45	5.764	***	Yes

### The mediation analysis

Hypothesis 4 (H4) tested the digital finance mediates the association among microfinance and ME development. This study followed the Awang et al. [11] identified technique to measure the mediation effect. In this study, the indirect effect is 0.26 (0.58 × 0.45 = 0.26), while the direct effect is 0.24. Hence, digital finance partially mediates the relationship among microfinance and ME development.

The Sobel test and bootstrap analysis were used to evaluate the mediation effects. The study used Sobel tests [50] to quantify digital finance's mediating impacts on microfinance services' associations with ME development. The findings show that digital finance plays an important role in mediating the association between microfinance and ME development (see Table 7).

**Table 7:** Results of mediation test

Path	Sobel tests Z	Bootstrapping effect	Results
MF – > MED	3.969***	0.24	Significant
MF – > DF – > MED	4.05***	0.26	Significant

***p < 0.001

One more method to deal with the sampling distribution of the constructs is to practice resampling, also known as “bootstrapping”. In methodological assessments, the bootstrapping procedure to get the mediating effect executes very well [50]. According to Awang et al. [11], bootstrapping was reliably the most influential. The results obtained are shown in Table 7. Therefore, hypothesis (H4) is accepted, as the consequence of the bootstrapping procedure confirmed the partial mediation occurred.

## Discussion

This study created an empirical microfinance model to examine and improve rural ME development as well as improve financial services in Malaysia. Micro-credit, micro-savings, and micro-insurance are the three financial services proposed in this study. It evaluated pertinent data and gave insights into MEs' perceptions of microfinance financial services in Malaysia. As a result, these constructs may be easily extended to various areas of the BOP market. This research also provides a technique for changing rural MEs' intent towards microfinance.

This conceptual model might help researchers who are investigating microfinance services in relation to rural micro enterprises and other BOP market customers. In addition, since other financial institutions have comparable features, this approach may be applied to them as well. This study conceptual model is unusual in that it incorporates digital finance as a mediating variable to modifying the microfinance services. In today's digital era, it offers researchers a unique conceptual model. As a result, microfinance services can contribute to rural MEs growth and considerably boost their entrepreneurial operations. Furthermore, digital finance has the potential to dramatically enhance the relationship between microfinance and ME development.

As a result, these variables may be integrated into the study model, which will help to better forecast microfinance services in the BOP market. Further discussion shows that this conceptual model might contribute to economic development research, which has been increasingly popular among academicians in recent years. This approach may also be applied for other financial goods and services used by customers from other market groups by adding digital finance services.

The findings indicated a substantial relationship between microfinance and rural ME development, which was consistent with prior research [15, 19, 20, 40]. In comparison to their competitors, rural MEs that used microfinance services evaluated the negative repercussions of a financial action extensively and tended to make more reasonable judgments. Furthermore, microfinance institutions that provide appropriate services may suffer the delightful ignorance effect, which indicates that they are aware of the implementation of their financial services but find it difficult to justify risk-related results [81].

It is worth noting that the mediating effect of digital finance has a substantial partial influence on microfinance and rural ME development, which is consistent with previous studies [5, 81]. Microfinance institutions should also think about integrating digital financial services, as it may assist them enhance connectivity, productivity, and service quality. The local government should establish laws that will help to speed up the process of collaboration between microfinance and MEs in rural areas.

## Conclusion

Microfinance is critical for improving the living conditions of poor MEs and alleviating poverty, since they require financial services to increase enterprise output and expand entrepreneurial intention. However, rural MEs are able to get microfinance services, they would face a variety of obstacles as well as opportunities, due to the misunderstandings among many stakeholders, microfinance has not acquired widespread acceptance. Irrational risk perceptions, in particular, prevent MEs from gaining access to microfinance institutions. As a result, this study investigates microfinance services in relation to the sustainable development of MEs in Malaysia's BOP market.

On the basis of BOP theory, the study developed a microfinance services model. The driving element in this model was digital finance, which served as a mediating variable. Data was collected from rural MEs using structured questionnaires, which were then statistically analyzed using AMOS-21. The findings of this study may be used directly by microfinance institutions and relevant government departments to improve microfinance services.

Our findings reveal that microfinance have a positive substantial influence on rural ME development, implying that a systematic financial services is required to enhance ME development in Malaysia's BOP market and to further stimulate their financing desires. Furthermore, it is critical to promote microfinance institutions' financial services in order to build rural MEs in Malaysia's BOP market, since microfinance's service qualities may cover the current gap and increase service availability, reliability, and effectiveness. Lastly, digital finance may considerably increase the favorable relationship between microfinance and the development of MEs as a mediating factor. As a result, the efficiency of digital finance may significantly lower ME transaction costs and promote good impressions of financial services of microfinance institutions.

Additionally, the digital finance innovation model has further encouraged the possibility of microfinance to play a bigger role in poverty reduction. Despite those effective microfinance models, it is important to emphasize that microfinance must continue to improve. Thus, it is important to enhance the institutional foundation for the development of microfinance services, which includes defining the legal status of microfinance, support government policy, and developing a microfinance service assessment system. Furthermore, the government's regulatory agencies requires an advanced innovative management system that reduces reluctance, and improves advantages. To increase participation, it is also necessary to lower entrance barriers for microfinance.

Furthermore, encouraging microfinance institutions to adopt a public-benefit attitude might expedite the adoption of financial services. It is critical for policymakers to develop selfless attitudes in order for microfinance services to achieve their social goals. One option is to use digital finance, which is critical in terms of improving service quality while reducing MEs' total costs. Because poor rural entrepreneurs have a limited knowledge, microfinance institutions are constantly revolutionizing their digital financial services in order to better fulfil the needs of BOP market consumers in Malaysia. To satisfy MEs' financial needs in today's digital environment, it is critical to combine the design of microfinance and digital financial services.

First limitation was the time constraint and COVID-19 travel ristriction, only a small quantity of data from Malaysian rural MEs was obtained. As a result, more research is needed to expand the sample size and conclude the social economic conclusions. Furthermore, the survey was conducted by the researchers, at a single moment in time; thus, future research should validate the conceptual model using longitudinal data.

Second, based on the setting of the BOP market in Malaysia, a unique conceptual model was presented. Researchers may use the conceptual model to evaluate the growth of rural MEs and microfinance after implementing innovative entrepreneurial activities. Future research might include aspects associated to innovation in the conceptual model. Furthermore, numerous empirical studies may be conducted to investigate the influence of microfinance on the performance of MEs.

Finally, the empirical findings of this study discovered that digital finance has a partial mediating relationship between microfinance services and rural ME development. In order to obtain novel results, the researchers might incorporate mediator(s) or moderator(s) in the existing framework.

## Data Availability

The data supporting the findings of this study are available within the article.
